# Evaluating the effectiveness and underlying mechanisms of incretin-based treatments for hypothalamic obesity: A narrative review

**DOI:** 10.1016/j.obpill.2024.100104

**Published:** 2024-02-24

**Authors:** Dionysios V. Chartoumpekis, Evagelia E. Habeos, Aristea Psilopanagioti

**Affiliations:** a Service of Endocrinology, Diabetology and Metabolism, Lausanne University Hospital and University of Lausanne, CH-1011, Lausanne, Switzerland; b Division of Endocrinology, Department of Internal Medicine, University of Patras, GR-26504, Patras, Greece

**Keywords:** Glp-1, Hypothalamus, Obese, GLP-1R, Semaglutide

## Abstract

**Background:**

Hypothalamic obesity represents a clinical condition within the broader spectrum of obesity that frequently eludes detection and appropriate diagnosis. This subset of obesity is characterized by a dearth of established predictive markers and a paucity of standardized therapeutic protocols. The advent and rising prominence of glucagon-like peptide-1 (GLP-1) receptor agonists in the obesity treatment landscape present novel therapeutic avenues for hypothalamic obesity management. Nonetheless, critical inquiries persist concerning the efficacy of GLP-1 receptor (GLP-1R) agonists in this context, particularly regarding their central mechanisms of action and specific impact on hypothalamic obesity.

**Methods:**

In this narrative review, we concentrate on analyzing research papers that delineate the detection and function of GLP-1 receptors across various hypothalamic and cerebral regions. Additionally, we examine clinical research papers and reports detailing the application of GLP-1 receptor agonists in treating hypothalamic obesity. Furthermore, we include a concise presentation of a clinical case from our unit for contextual understanding.

**Results:**

Currently, the clinical evidence supporting the efficacy of GLP-1 receptor agonists in hypothalamic obesity, as well as the diverse characteristics of this obesity subtype, remains insufficient. Preliminary data suggest that GLP-1R agonists might offer an effective treatment option, albeit with variable outcomes, particularly in younger patient cohorts. From a mechanistic perspective, the presence of GLP-1 receptors in various hypothalamic and broader brain regions potentially underpins the efficacy of GLP-1R agonists, even in instances of hypothalamic damage. Nevertheless, additional research is imperative to establish the functional relevance of these receptors in said brain regions.

**Conclusion:**

GLP-1R agonists represent a potential therapeutic option for patients with hypothalamic obesity. However, further clinical and basic/translational research is essential to validate the efficacy of these drugs across different presentations of hypothalamic obesity and to understand the functionality of GLP-1R in the diverse brain regions where they are expressed.

## Graphical abstract

**Figure:**
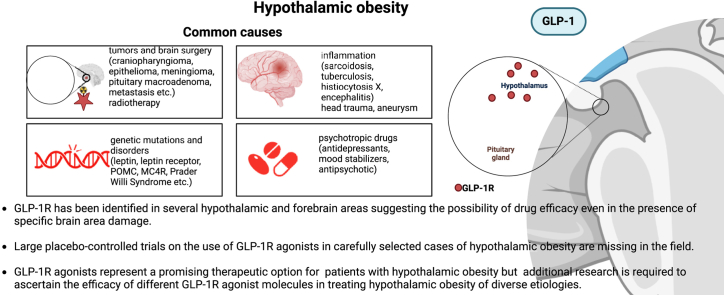

## 1 Introduction

**Fig. 1:**
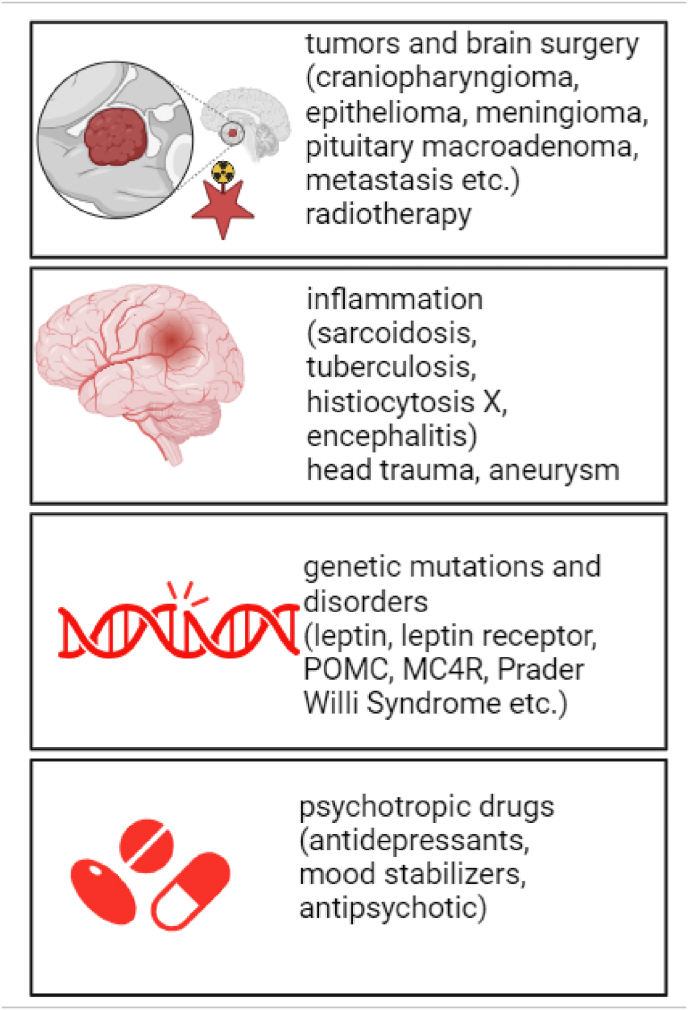
Some causes of hypothalamic obesity (POMC; proopiomelanocortin, MC4R; melanocortin-4 receptor). The relevant information used to generate this figure is found in references 3-7. Created with biorender.com.

The hypothalamus plays a pivotal role in regulating body weight and energy homeostasis, a conclusion supported by a multitude of experimental studies in the past [1]. This assertion is further corroborated by the emergence of monogenic obesity syndromes, which are often attributed to mutations in genes predominantly expressed in the hypothalamus [2]. Additionally, there is a notable incidence of obesity in patients following extensive suprasellar surgical procedures in the brain, leading to the classification of ‘hypothalamic obesity.' Some of these patients develop also obesity even before the surgery. Suprasellar tumors, most commonly craniopharyngiomas consist a classic cause of hypothalamic obesity [3]. Over time, the definition of this term has expanded to encompass various hypothalamic pathologies that may precipitate obesity (reviewed in Ref. [4] and briefly depicted in Fig. 1). The mechanisms that lead to hypothalamic obesity are not fully elucidated but they include hyperphagia, defects in satiety [5] that are regulated by hypothalamic areas such as ventromedial hypothalamus, paraventricular nuclei, arcuate nucleus and the lateral hypothalamic area.

Even though overall hypothalamic obesity is a relatively rare cause of obesity *per se*, such obesity cases are more often recognized the last decades because of the higher sensitivity to investigate the origins of body weight gain and also of the better post-surgery follow-up of patients with suprasellar tumors. Hypothalamic obesity is also difficult to manage successfully in outpatient obesity clinics and various drugs and bariatric surgery had been used with variable results [6]. Given the increasing popularity and coverage by health insurance policies of incretin-based medications for obesity, most notably glucagon-like peptide 1 (GLP-1) receptor agonists and double GLP-1/glucose-dependent insulinotropic polypeptide (GIP) receptor agonists, the use of these medications in hypothalamic obesity has increased and they have shown variable results ranging from a stabilization of weight gain to significant weight loss [7]. As the main effect of incretin-based therapies regarding the weight loss lies on hypothalamus, there are concerns regarding the efficacity of these drugs in the case that regions of the hypothalamus are affected by a surgery in the area or if intracellular signaling in hypothalamus is affected by mutations in cases of monogenic obesity. Herein we summarize the existing information regarding the efficacity of incretin-based therapies in cases of hypothalamic obesity providing also the example of a relevant clinical case and we review the current knowledge regarding the different levels of actions of GLP-1 agonists in various areas of hypothalamus.

## 2 Localization of GLP-1 receptors (GLP-1R) and different levels of action of GLP-1 in hypothalamus

In the physiological control of food intake, rodent studies support a role for multiple GLP-1R-expressing regions in the central nervous system, both hypothalamic and hindbrain. However, little information is available about the physiological role of GLP-1R and endogenous GLP-1 in feeding and body weight control in humans. The available information in the literature regarding the localization of GLP-1R in rats and humans is summarized in Table 1 and is discussed in the following subsections.

**Table 1:** Semi-quantitative comparison of GLP-1R immunoreactivity in rat and human hypothalamic and basal forebrain nuclei, at mRNA and protein level.

Rat studies	
Region	GLP-1R mRNA (ISH)^a^
Paraventricular nucleus	++++
Supraoptic nucleus	+++
Arcuate nucleus	++++
Lateral hypothalamic area	+
Perifornical region	na
Ventromedial hypothalamic nucleus	−
Dorsomedial hypothalamic nucleus	+
Tuberomammillary nucleus	na
Medial preoptic area	++
Suprachiasmatic nucleus	na
Mammillary nucleus	+
Basal nucleus	na
Diagonal band	+
Bed nucleus of the stria terminalis	+

Human studies		
Region	GLP-1R mRNA (ISH)^b^	GLP-1R protein (IHC)^c^
Paraventricular nucleus	++	++
Supraoptic nucleus	++	+++
Infundibular (arcuate) nucleus	+	++
Lateral hypothalamic area	+	++
Perifornical region	+	++
Ventromedial hypothalamic nucleus	++	++
Dorsomedial hypothalamic nucleus	++	+
Tuberomammillary nucleus	++	++
Medial preoptic nucleus	+	++
Suprachiasmatic nucleus	+	−
Mammillary nucleus	+	++
Basal nucleus	++	++
Diagonal band	++	++
Bed nucleus of the stria terminalis	+	++

Rat ISH data from references 11 and 12. Human ISH data from references 17 and 19.

a Overview of areas in the rat hypothalamus containing GLP-1R mRNA-expressing cells, from low (+) to high (++++) density of labeled cells. na, no data available.

b In situ hybridization (ISH): ++, the majority of cells are positive and staining intensity is high; +, the majority of cells are positive and cells show a moderate to strong signal; −, no staining.

c Immunohistochemistry (IHC): +++, >50% of cells exhibit high immunoexpression; ++, >50% of cells exhibit moderate immunoexpression; +, >50% of cells exhibit low immunoexpression; −, no staining.

### 2.1 Rat studies

Evidence from animal studies suggests that central GLP-1 is a physiological mediator of satiety. Hypothalamus and hindbrain are brain regions of primary importance in the GLP-1R-mediated regulation of feeding behavior [[8], [9], [10]]. In rodent hypothalamus, both the paraventricular nucleus (PVN) and the arcuate nucleus (ARC) are the two hypothalamic nuclei with the highest density of GLP-1Rs [11,12]. Intracerebroventricular GLP-1 administration induces c-fos, a marker of neuronal activation, exclusively in the paraventricular hypothalamic nucleus and elicits a powerful anorectic response [11]. It is generally believed that rodent PVN is the primary area at which endogenous brain-derived GLP-1 exerts its anorexigenic/satiating effects [11]. PVN infusion of GLP-1-(7-36) amide suppressed feeding in rats [13]. In the arcuate nucleus, GLP-1R agonist is internalized in neurons expressing CART and proopiomelanocortin (POMC). In rats with diet-induced obesity, long-term administration of a GLP-1R agonist regulates neuronal expression of cocaine- and amphetamine-regulated transcript (CART) and neuropeptide Y (NPY)/Agouti-related protein (AgRP) (increased CART and reduced the expression of NPY, AgRP) in the arcuate nucleus and decreases food intake and body weight [14]. The lateral hypothalamic area (LH) is a key reward-control region in the brain, suggested to be the interface between the homeostatic and hedonic feeding behavior. In rats, LH was shown to mediate weight loss and the reward inhibitory effects of exogenous GLP-1R activation and to be a crucial site for motivated and ingestive behavior suppression driven by endogenous GLP-1R activation [15]. In rat lateral hypothalamic area, selective GLP-1R activation induced a robust food intake and body weight reduction. Although acute pharmacological blockade increased food motivation but only in male rats, chronic (genetical) GLP-1R knockdown, selectively in the LH, markedly elevated ingestive behavior and increased body weight accompanied with fat mass doubling in male and female rats. These findings, identify the LH GLP-1R as an indispensable element of normal body weight homeostasis and food reward behavior [15].

### 2.2 Human studies

A few studies have presented experimental evidence for the localization of GLP-1 receptor mRNA and protein in human hypothalamus, using *in situ* hybridization techniques [16,17] and immunohistochemistry [[18], [19], [20]]. In most of these studies, GLP-1R showed a particularly heterogenous mRNA and protein expression with strong interindividual variations. Interestingly, a decreased expression of GLP-1R was reported in the lateral hypothalamic area in patients with a body mass index (BMI) ≥ 25 kg/m^2^ [19] and in the paraventricular and infundibular (arcuate) hypothalamic nuclei in patients with type 2 diabetes mellitus [17]. Furthermore, in the human hypothalamus, GLP-1R was extensively colocalized with the anorexigenic and anti-obesogenic neuropeptide nucleobindin-2/nesfatin-1 in the paraventricular, supraoptic, and infundibular nuclei, in the lateral hypothalamic area, and in basal forebrain nuclei [19] and sporadically colocalized with energy balance-related neuropeptides, such as NPY, AgRP, and POMC, in the infundibular nucleus [17]. In addition, GLP-1R, was coexpressed with glucose transporter isoform (GLUT-2) and glucokinase (GK) mRNAs in the same cells, mainly of the ventromedial and arcuate nuclei of the hypothalamus [16]. GLP-1R has been also detected in human cholinergic basal forebrain nuclei [17,19], which are known to degenerate in Alzheimer's disease. Although the functional importance of GLP-1R presence in basal forebrain nuclei remains to be determined, it should be noted that GLP-1R agonism may prevent cholinergic basal forebrain nuclei degeneration [21].

In the human hypothalamus, GLP-1R was localized in neurons [[17], [18], [19]]. Although astrocytes have been proposed to contribute to the transduction of anorectic GLP-1R-dependent signals in rats [22], studies in the human hypothalamus have not described glial expression of GLP-1R or colocalization of GLP-1R with the astrocytic marker glial fibrillary acidic protein (GFAP) in hypothalamic nuclei [[16], [17], [18], [19]]. Furthermore, GLP-1R-immunoreactive fibers are widely distributed in the human hypothalamus [18,19].

GLP-1R mRNA and protein expression was confirmed by functional expression using *in situ* ligand binding with ^125^I-GLP-1(7–36) amide in several human brain areas including the hypothalamus [16].

A small number of studies have examined the potential neural mechanisms activated by GLP-1 receptor agonism in the human brain. Using positron emission tomography, Alvarez et al. reported that the i.v. administration of GLP-1(7-36) amide significantly reduced cerebral glucose metabolism in human hypothalamus and brainstem [16]. Using functional MRI, in 48 patients with diabetes and obesity as well as normoglycemic subjects with obesity and lean individuals, van Bloemendaal L et al. found increased brain responses to food pictures in appetite- and reward-related brain regions, such as the insula and amygdala, after intravenous administration of the GLP-1R agonist exenatide [23]. Furthermore, GLP-1R activation reduced brain responses to food cues as well as food intake in normoglycemic individuals with obesity and patients with type 2 diabetes and obesity in the insula, amygdala, putamen, and orbitofrontal cortex, brain areas involved in eating behavior and reward.

According to Farr et al., GLP-1R agonist liraglutide decreased activation of the parietal cortex in response to highly desirable food and decreased activation in the insula and putamen [18]. However, activation of the hypothalamus with GLP-1R analogues was not observed in humans [18]. This may be due to a technical limitation of functional magnetic resonance imaging (MRI), as the hypothalamus is difficult to detect because of its small size and close proximity to the sinuses, which create artefacts [24]. Importantly, among individuals treated subcutaneously with the GLP-1R agonist exenatide, those who respond with weight loss, show significantly higher connectedness, i.e. higher influence of the hypothalamus on the rest of the brain, a finding indicative of the importance of the hypothalamic response for the effect of exenatide on food intake and body weight in humans [25]. The hypothalamus, coordinates autonomic neural, including vagal, and hormonal signals about energy homeostasis [26]. It has been suggested that vagal innervation may be important in mediating the effects of GLP-1 on food intake in male patients [27].

## 3 GLP-1 receptor agonists in the treatment of hypothalamic obesity

### 3.1 Information from the bibliography about GLP-1R agonists use in hypothalamic obesity

It is always difficult to predict the treatment response to GLP-1RA in patients with obesity in general and even more to patients with hypothalamic obesity. Earlier reports have shown that a treatment by GLP-1 analogues (exenatide) is efficient in adult patients with hypothalamic obesity leading to a weight loss that varies from 5 to 10% of initial body weight after 4-12 months of treatment [28]. A randomized clinical trial with a weekly exenatide injection during 36 weeks in 42 patients between the age of 10–25 years with hypothalamic obesity mainly due to a craniopharyngioma did not show a significant difference in body weight change (−3.6 kg) but showed a clear trend for reduced fat mass in treated patients [29]. The variability in the response to GLP-1 analogues of patients with hypothalamic obesity and the mediocre effect on body weight could be due to the fact that these patients were mostly young and during their development. A naïve explanation would also be that the more damage is done to the hypothalamus after surgery the harder it would be to observe a response to GLP-1RA given that less hypothalamic nuclei would be available for the GLP-1RA to act upon. Given that it is not easy to really quantify the damage incurred to the hypothalamus after a surgery, such studies are scarce. The efficacity of an GLP-1RA (exenatide 2 mg per week for 36 weeks) to reduce significantly the body fat content and improve or ameliorate hypothalamic obesity (trend for a decrease in BMI) was shown in a randomized clinical trial of 42 adolescents and young adults [30]. An interesting approach was implemented by Perez et al. where 35 adolescent subjects were categorized using an MRI-based hypothalamic lesion score (HLS), after surgery for craniopharyngioma [31]. It was found that patients with higher HLS scores, and particularly more extensive damage at the mammillary body area, had higher adiposity (percentage of body fat) at baseline and showed also greater reduction in body fat percentage following a treatment with exenatide 2 mg per week during 36 weeks [31]. Thus, it seems that the extent of the hypothalamic damage does not impede the efficacity of GLP-1RA. It is possible that a more extensive damage of hypothalamus may limit the ligand sites for the endogenous GLP-1 but the remaining sites in hypothalamus (described extensively in section 2) and other central nervous system areas may be more responsive to the exogenous ligands (GLP-1RA).

It is true that there are not enough clinical trials so far with hypothalamic obesity patients using the newest GLP-1RA such as liraglutide and semaglutide. A placebo-controlled trial using liraglutide 3 mg per day on Prader-Willi syndrome patients (adolescents and children) did not show a significant difference in BMI change after a total period of 52 weeks [32]. The only significant difference seen was the reduction of hyperphagia score in the adolescent population. The use of semaglutide at a dose of 2.4 mg per week was highlighted in a case report of a 16-year old boy without diabetes who developed obesity after the resection of a craniopharyngioma [33]. A 6-month semaglutide treatment resulted in a 31 kg (about 25%) weight loss.

### 3.2 A case report of semaglutide treatment in a female patient with type 2 diabetes and hypothalamic obesity post-craniopharyngioma surgery

**Fig. 2:**
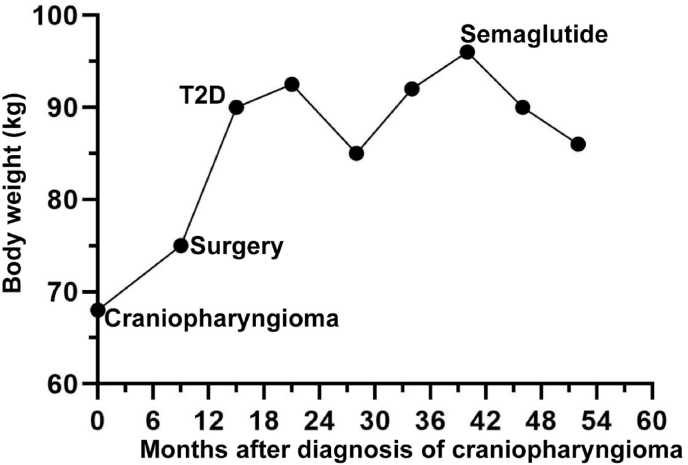
Body weight evolution of a 45 years old woman with hypothalamic obesity due to an adamantinomatous craniopharyngioma which was surgically excised (“Surgery”). T2D: denotes the timepoint that a diagnosis of type 2 diabetes mellitus was made. Semaglutide: denotes the timepoint when a treatment with semaglutide injections was established (0.25 mg/week for the first month, 0.5 mg/week for the second month and 1 mg/week thereafter.

From our experience in the division of Endocrinology of University of Patras, Greece we had also tried a treatment with semaglutide in the context of type 2 diabetes in a 45 year-old female patient who was diagnosed with an adamantinomatous craniopharyngioma with an initial body weight of 68 kg (BMI = 24.1 kg/m^2^). The patient showed a significant weight gain after the surgery arriving at a body weight of 90 kg (BMI = 31.9 kg/m^2^) 6 months after the surgery and was diagnosed at that time with a type 2 diabetes (Fig. 2). She showed no signs of hypercortisolism given that she was being treated with hydrocortisone for hypopituitarism after the surgery. Initially she was started on metformin 1000 mg twice daily and with some lifestyle interventions (increased physical activity and dietary support by a professional nutritionist) she was able to lose weight arriving at 85 kg (BMI = 30.1 kg/m^2^) 13 months after the T2D diagnosis. Afterwards, she regained the body weight lost and arrived at the maximum body weight of 96 kg (BMI = 34 kg/m^2^). That's when a treatment with an once weekly injection of semaglutide was initiated with 0.25 mg for the first 4 weeks, 0.5 mg for the weeks 5–8 and at 1 mg afterwards. After having been for 12 months on semaglutide she has lost 10 kg (10.4% body weight loss since the initiation of semaglutide) arriving at a body weight of 86 kg (BMI = 30.5 kg/m^2^) (Fig. 2). It is currently planned to increase gradually the dose of semaglutide up to 2 mg per week.

## 4 Conclusions and perspectives

The recent surge in the use of GLP-1R agonists as an effective obesity treatment marks a significant advancement in their application for hypothalamic obesity as well. Given that the primary mechanism of these drugs in inducing weight loss is centered in the hypothalamus, it becomes crucial to ascertain their functionality in cases of hypothalamic obesity, particularly when hypothalamic regions are compromised due to tumors, surgical interventions, or inflammation. This review compiles, to the best of our knowledge, existing data on the localization of GLP-1R across various hypothalamic and cerebral regions, facilitating their potential therapeutic action. Notably, GLP-1R has been identified in several hypothalamic and forebrain areas (Table 1), suggesting the possibility of drug efficacy even in the presence of specific brain area damage. Nonetheless, these findings should be interpreted with caution due to potential issues such as lack of adequate controls and the possibility of false-positive results from antibody misuse [34]. We have made every effort to include in this review only manuscripts that have used all proper controls. Moreover, the presence of GLP-1R does not convey the functionality of the receptor. Functional imaging such as fMRI or GLP-1 based PET-CT (exendin-3) [35] could be useful in detecting the activation of certain brain and hypothalamus areas with limitations including the very small size of hypothalamus.

Nevertheless, the best metric of the efficiency of a GLP-1R agonist in cases of hypothalamic obesity is the weight loss induced by actually treating a patient with it. It is evident that large placebo-controlled trials on the use of GLP-1R agonists in carefully selected cases of hypothalamic obesity are missing in the field. Some studies using exenatide [[28], [29], [30]] and liraglutide [32] in mainly adolescent-young patients that were reviewed herein showed variable results regarding weight loss but with a favorable effect on fat mass content and hyperphagia. Further studies are needed using liraglutide and other newer molecules such as semaglutide so as to evaluate them in patients with carefully identified hypothalamic obesity of various origins (Fig. 1). The initial experiences of our groups and others with the use of semaglutide in mainly adult patients with hypothalamic obesity are promising ([33] and Fig. 2). Other drugs of the incretin family such as tirzepatide (dual GLP-1R and GIPR agonist) need to be tested in cases of hypothalamic obesity along with other drugs such as setmelanotide (MC4R agonist) with proven efficacy on cases of monogenic obesity [36].

• Vigilant identification of hypothalamic obesity within obesity outpatient clinics is imperative to ensure appropriate patient management and follow-up.
• GLP-1R agonists represent a promising therapeutic option for patients with hypothalamic obesity but additional research is required to ascertain the efficacy of different GLP-1R agonist molecules in treating hypothalamic obesity of diverse etiologies.
• Advancements in diagnostic techniques to assess hypothalamic damage may enable more personalized treatment approaches and enhance prognostic accuracy for individual obesity cases. This, in turn, could facilitate more informed and evidence-based clinical decision-making. Furthermore, continued basic and translational research into the roles and mechanisms of GLP-1R and their agonists at the physiological and molecular levels is essential to drive progress in this field.

## Ethical review

This review article contains anonymized clinical data of a real patient who is used as an example. The patient has provided an informed written consent for the use of her anonymized clinical data.

## Source of funding

This manuscript is not funded by any specific grant from funding agencies in the public, commercial, or not-for-profit sectors. DVC is supported by a 10.13039/501100006390University of Lausanne grant (Bourse de relève academique).

## Declaration of artificial intelligence (AI) and AI-assisted technologies

During the preparation of this work the authors did not use AI.

## CRediT authorship contribution

DVC and AP conceptualized the manuscript. DVC, EEH and AP conducted the literature review and wrote the first draft. DVC and AP reviewed the final manuscript and approved the final submission and publication.

## Declaration of competing interest

All the authors listed have approved the manuscript and have no conflicts of interest on this paper.
